# BET Inhibition Improves NASH and Liver Fibrosis

**DOI:** 10.1038/s41598-018-35653-4

**Published:** 2018-11-22

**Authors:** Sarah A. Middleton, Neetu Rajpal, Leanne Cutler, Palwinder Mander, Inmaculada Rioja, Rab K. Prinjha, Deepak Rajpal, Pankaj Agarwal, Vinod Kumar

**Affiliations:** 1 Computational Biology, GSK, 1250 S. Collegeville Road, UP12-100, Collegeville, PA 19426-0989 USA; 2 Quantitative Pharmacology, Immuno-Inflammation Therapy Area, Medicines Research Centre, GSK, Gunnels Wood Road, Stevenage, SG1 2NY UK; 3 Epigenetics DPU, Oncology Therapy Area, Medicines Research Centre, GSK, Gunnels Wood Road, Stevenage, SG1 2NY UK

## Abstract

Non-alcoholic fatty liver disease (NAFLD) is a leading form of chronic liver disease with large unmet need. Non-alcoholic steatohepatitis (NASH), a progressive variant of NAFLD, can lead to fibrosis, cirrhosis, and hepatocellular carcinoma. To identify potential new therapeutics for NASH, we used a computational approach based on Connectivity Map (CMAP) analysis, which pointed us to bromodomain and extra-terminal motif (BET) inhibitors for treating NASH. To experimentally validate this hypothesis, we tested a small-molecule inhibitor of the BET family of proteins, GSK1210151A (I-BET151), in the STAM mouse NASH model at two different dosing timepoints (onset of NASH and progression to fibrosis). I-BET151 decreased the non-alcoholic fatty liver disease activity score (NAS), a clinical endpoint for assessing the severity of NASH, as well as progression of liver fibrosis and interferon-γ expression. Transcriptional characterization of these mice through RNA-sequencing was consistent with predictions from the CMAP analysis of a human NASH signature and pointed to alterations in molecular mechanisms related to interferon signaling and cholesterol biosynthesis, as well as reversal of gene expression patterns linked to fibrotic markers. Altogether, these results suggest that inhibition of BET proteins may present a novel therapeutic opportunity in the treatment of NASH and liver fibrosis.

## Introduction

Nonalcoholic fatty liver disease (NAFLD) is one of the most common causes of chronic liver disease in Western countries^1^. NAFLD encompasses a spectrum of fatty liver diseases observed in individuals that do not consume excessive alcohol, ranging from simple steatosis to steatosis accompanied by hepatocyte ballooning and inflammation (referred to as nonalcoholic steatohepatitis, NASH) and fibrosis^2^. The estimated global prevalence of NAFLD is now 24%, with rates over 30% in South America and the Middle East, while the prevalence of NASH is estimated to be between 3–16%^3–5^. Patients with NASH are more likely to progress to cirrhosis, liver failure, and hepatocellular carcinoma (HCC), and have an estimated 10-fold increased risk of liver-related death compared to patients with only simple steatosis^3^. NASH is now the second most common reason for liver transplantation in the US, after chronic hepatitis C^3^.

A key characteristic of NASH is that the fat accumulation is accompanied by inflammation and hepatocellular damage. The mechanism by which simple steatosis progresses to NASH is currently not well understood, but several key risk factors have been identified, including obesity, diabetes, hyperlipidemia, and genetic predisposition^6^. Several therapeutic intervention strategies targeting various steps in the development of steatosis and steatohepatitis^7^ have been evaluated, but there are currently only limited treatment options for NASH. Due to the growing incidence of this disease, there is an increasing need to find a suitable pharmacotherapy.

The Bromodomain and Extra-Terminal domain (BET) family of epigenetic proteins (BRD2, BRD3, BRD4 and BRDT) are “readers” acetylated histone and non-histone proteins^8,9^ that play an important role in regulating the co-activation and co-expression of many pro-inflammatory genes by modulating promoter and enhancer activity^10–12^. The BET family of proteins have been the subject of great interest for drug discovery and development due to their potential as therapeutic targets for diseases ranging from cancer to inflammation, neurological disorders, and cardiovascular disease^13–16^. Several small molecule inhibitors of BET have progressed into the clinic^17^, and protection against liver fibrosis as well as reversal of fibrotic progression has been reported in a chemically induced mouse model following BET inhibition^14,18^.

Given the existing evidence relating BET and liver fibrosis, plus an association via connectivity map (CMAP)^19^ to NASH which we report here, we decided to further validate this mechanism. As part of this effort, we investigated the role of a small-molecule inhibitor of the BET family of proteins, GSK1210151A (I-BET151)^20,21^, in a pre-clinical mouse NASH STAM model^22^ to assess its efficacy against NASH and hepatic fibrosis. In the STAM model, streptozotocin (an N-acetyl-β-D-glucosaminidase inhibitor; STZ) is administered neonatally to produce mild systemic inflammation and hyperglycemia, which is then followed by a high-fat diet challenge starting at postnatal week four. The dose of STZ is designed to produce a partial impairment of insulin secretion, and the resultant phenotype is similar to advanced type 2 diabetes (T2D) where beta cells are functionally exhausted and fail to secrete insulin at normal levels^23^. The NASH STAM model is widely used primarily because it recapitulates the full spectrum of human NAFLD ranging from steatosis to NASH and hepatic fibrosis. In addition, the histological phenotypes observed in this model are like those seen in human clinical samples^24^, which allows the same scoring system (NAFLD activity score; NAS) to be used to assess the severity of the disease. In this study, we found that therapeutic intervention with I-BET151 resulted in significant reduction in NAS and improvement in liver fibrosis scores in the STAM mice. Transcriptional characterization of the treated mice pointed to alterations in interferon signaling in particular, suggesting a mechanism contributing to the observed histological improvements following BET inhibition. Overall, the results from our study suggest that BET inhibition may represent a novel therapy for the treatment of NASH and other fibrotic liver diseases.

## Results

### Identification of a link between BET inhibition and human NASH by Connectivity Map analysis

An *in silico* bioinformatics approach (CMAP analysis^19^) was utilized to identify potential candidate targets for the treatment of NASH by exploring transcriptional expression profiles associated with an existing GSK compound collection. Briefly, CMAP is a transcriptional expression database that catalogs gene signatures elicited by chemical perturbagens. By employing this approach, the generated data allows a comparison of gene expression profiles following compound treatment of cell lines to that of carefully curated expression signatures of diseased tissue from patients or models. When an inverse correlation is established through computational analysis between a compound-generated signature and a disease signature, an evaluation is carried out to assess whether the underlying compound mechanisms are therapeutically relevant for disease resolution, on a case by case basis. We created profiles for over 400 GSK compounds, including multiple BET inhibitors, across several cell lines as part of a systematic drug repurposing project. These profiles were compared to over 6000 disease signatures assembled from both public-domain and proprietary sources (NextBio^TM^). During the computational analysis, we identified significant inverse correlation relationships between BET compounds and gene expression signatures of steatosis, liver fibrosis, and patient-derived NASH (Supplementary Table S1), suggesting that BET compounds may have utility for treatment of these conditions.

### Mouse STAM NASH Model

**Figure 1:**
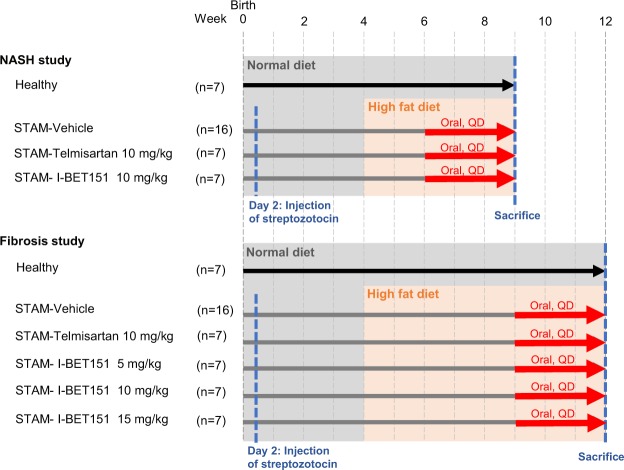
Design of the NASH and fibrosis studies using the NASH STAM model. Neonatal male mice in the treatment groups were injected with STZ on day 2 to induce a diabetic state. Mice were fed a normal diet until week 4, at which point the treatment groups were switched to a high fat diet. Mice were dosed with either vehicle, Telmisartan, or I-BET151 once a day (*quaque die*, QD) from week 6–9 for the NASH study or week 9–12 for the fibrosis study.

To explore this hypothesis further, we tested a small molecule BET inhibitor, I-BET151, in a STAM mouse model^22^ at two distinct time windows to evaluate its efficacy on early NASH (“NASH study”, week 6–9) and during fibrosis onset (“fibrosis study”, week 9–12) (Fig. 1). The Mouse STAM model offers the advantage of monitoring the natural progression from fatty liver to NASH and fibrosis in a relatively controllable fashion. It defines the onset of NASH (with hepatocyte fat accumulation and inflammation) and fibrosis as occurring between 6–8 weeks and 9–12 weeks respectively; these transition stages have been well characterized in the model and are based on the macroscopic and histopathology characteristics resembling human NASH and fibrosis. For the NASH study, mice were orally dosed with I-BET151 for 3 weeks, starting at 6 weeks of age. Similarly, for the fibrosis study, I-BET151 was orally administered for 3 weeks, this time starting at 9 weeks of age. Telmisartan (10 mg/kg), an angiotensin receptor blocker which has been shown to improve NAS in human NASH patients^25^, was included as a positive control in both the studies.

### Body/Liver Weight and Serum/Liver Biomarkers

Details on body/liver weight and serum/liver biomarkers for each study can be found Supplementary Tables S2, S3 and S4. Overall, I-BET151 treatment was well tolerated in the STAM model and showed no deleterious effects on body and liver weight, and liver function. Body weights increased gradually following I-BET151 administration in both the NASH and fibrosis studies, but are moderately lower compared to the control/healthy group. Liver weights and liver-to-body weight ratios showed no significant changes upon treatment.

Serum ALT levels are significantly higher in the vehicle and the I-BET151 treatment groups compared to healthy in both NASH and fibrosis studies. Interestingly, serum AST levels showed no marked changes in healthy, vehicle and drug-treated groups in the NASH study, but trend higher for the I-BET151 treated group in the fibrosis study. Insulin levels in the vehicle and I-BET151 treatment groups are significantly lower compared to healthy group, in both the NASH and fibrosis studies. Serum cholesterol levels in the I-BET151 treatment groups show no marked differences compared to vehicle, but increased significantly compared to the healthy group. Serum triglycerides levels are higher for the I-BET151 treatment groups in the fibrosis study.

### NASH Study: Glucose, Liver Histopathology, and NAFLD Activity Scores

**Figure 2:**
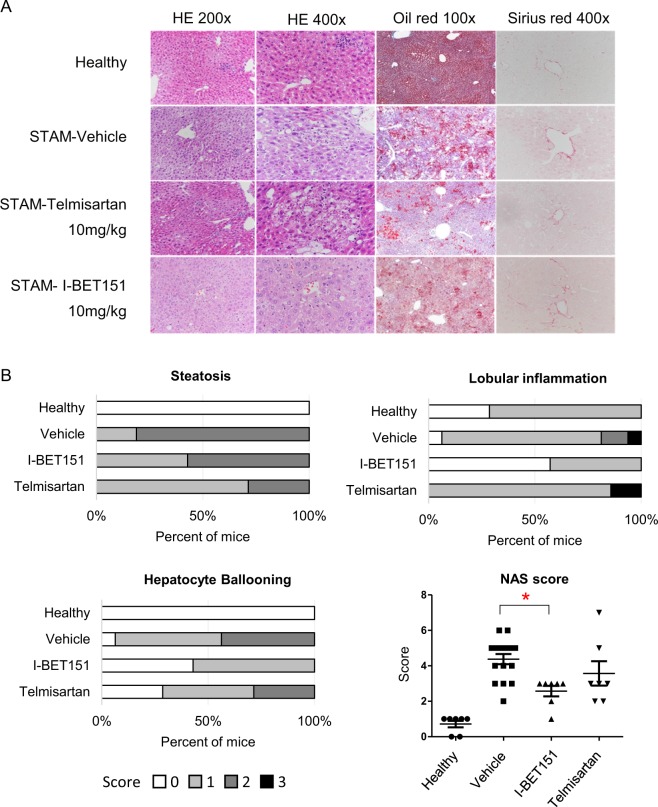
Reduction of NAS in STAM mice following I-BET151 treatment. **(A)** Representative H&E stained (200x and 400x), Oil-red (100x) stained, and Sirius-red (400x) stained liver sections from healthy, vehicle, Telmisartan, and I-BET151 treated NASH STAM mice collected at week 9, showing the histological features of the NASH STAM model at this timepoint for the different groups. **(B)** Comparison of NAS from mouse liver specimens in 9 week old mice from each treatment group. Individual components of the NAS, which include steatosis, lobular inflammation, and hepatocellular ballooning degeneration, are shown for each of the cohorts. Interpretation was performed by a pathologist blinded by treatment group. Telmisartan, n = 7; I-BET151, n = 7; Vehicle, n = 16; Healthy, n = 7. *p = 0.0032 vs Vehicle Control (Wilcoxon test).

In the NASH study, glucose levels increased significantly in the vehicle-treated STAM group compared to healthy group, suggesting that the NASH model animals may have developed islet β-cell dysfunction^26^. However, in the group that received I-BET151 treatment, glucose levels were significantly reduced relative to the vehicle (Supplementary Table S2). At 9 weeks of age, liver sections from the vehicle group exhibited severe micro- and macro-vesicular fat deposition with predominantly macro-vesicular fat, as well as hepatocellular ballooning and inflammatory cell infiltration. Representative photomicrographs of the H&E stained liver sections are shown in Fig. 2A and Supplementary Fig. S1. Consistent with these observations, NAS increased significantly from 0.71 ± 0.18 in the healthy group to 4.38 ± 0.29 in the vehicle group (*p* = 3.3 E-10, Wilcoxon test; Fig. 2B) based on the NASH CRN scoring system^27^. Mice treated with I-BET151 showed a significant improvement in NAS compared to the vehicle-treated group, reducing the scores to 2.57 ± 0.30 (*p* = 0.0023, Wilcoxon test; Fig. 2B), with both lobular inflammation and hepatocyte ballooning improving by over 50% in the I-BET151-treated mice. A follow up dose-response study also showed a similar trend, where mice treated with I-BET151 showed a statistically significant improvement the NAS scores compared to the vehicle group at doses of 5 and 10 mg/kg (*p* < 0.05, Wilcoxon test; Supplementary Table S5).

### Fibrosis study: Glucose, Liver Histopathology, and Measurement of Fibrosis

**Figure 3:**
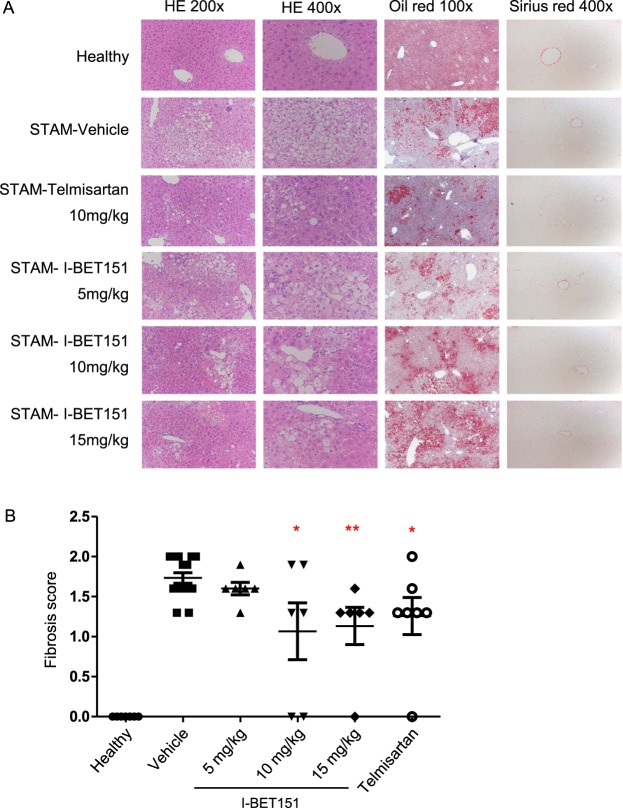
Reduction of fibrosis in STAM mice following I-BET151 treatment. **(A)** Representative H&E stained (200x and 400x), Oil-red (100x) stained, and Sirius-red (400x) stained liver sections from healthy, vehicle, Telmisartan, and I-BET151 treated NASH STAM mice collected at week 12. **(B)** Histopathology assessment of fibrosis scores from mouse liver specimens obtained at 12 weeks of age following I-BET151 and Telmisartan treatment between weeks 9–12. The scoring was determined based on the NASH CRN scoring. Data are expressed as means ± SEM. Asterisks denote significant changes compared to Vehicle (*p < 0.05, **p < 0.01; Wilcoxon test).

In the fibrosis study, both glucose and insulin levels were significantly reduced following I-BET151 treatment relative to the vehicle (Supplementary Table S3). Sirius red staining of livers at 12 weeks showed various degrees of a moderate perisinusoidal collagen deposition starting from the central area and extending into the hepatic lobules, including in the vehicle group (Fig. 3A and Supplementary Fig. S2). The percentage of fibrosis area (Sirius red-positive area) significantly increased in the vehicle-treated group compared to the healthy animals (*p* < 0.001, Wilcoxon test; Fig. 3B), but decreased in the I-BET151 treatment groups relative to the vehicle, with mice dosed at the highest concentrations (10 and 15 mg/kg) showing a statistically significant reduction in fibrosis score when compared to the vehicle group (*p* = 0.05, 0.0041, respectively, Wilcoxon test; Fig. 3B).

### Expression of inflammatory and collagen/fibrosis markers

**Figure 4:**
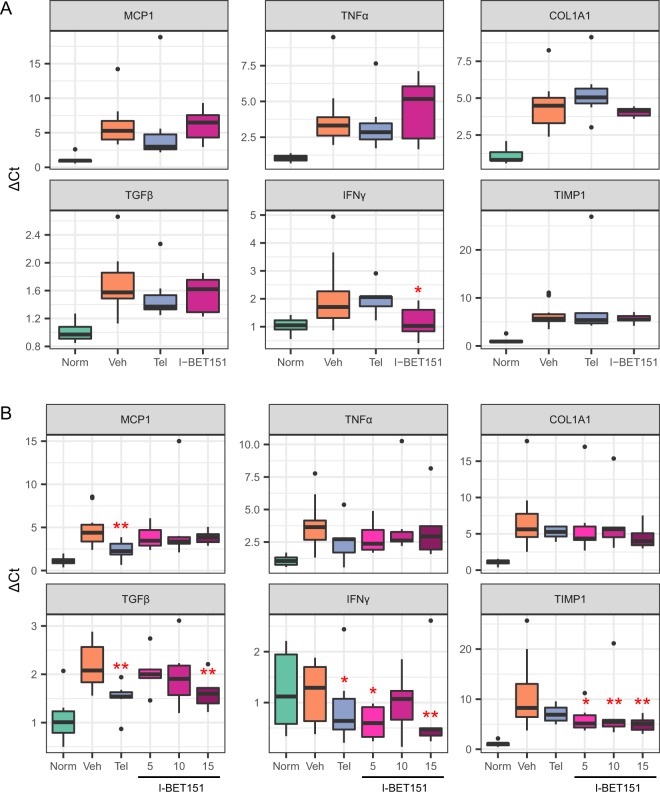
Gene expression of inflammatory and fibrosis markers from RT-qPCR. Expression of marker genes in Healthy (Norm), STAM-Vehicle (Veh), STAM-Telmisartan (Tel), and STAM-I-BET151 groups in the **(A)** NASH and **(B)** fibrosis studies. I-BET151 concentrations are shown in mg/kg. ΔCt was computed relative to Rplp0 reference. Asterisks denote significant changes compared to Vehicle (*p < 0.05, **p < 0.01, one way ANOVA followed by Fisher LSD test). Outliers (>Mean ± 2*SD) are shown but were excluded during the statistical analysis.

We analyzed mRNA expression levels of the inflammatory cytokines tumor necrosis factor-α (TNF-α), monocyte chemoattractant protein-1 (MCP-1) and interferon-γ (IFN-γ), as well as the fibrosis markers TIMP metallopeptidase inhibitor 1 (TIMP1), collagen type 1 alpha 1 (COL1A1), and transforming growth factor-β (TGF-β) in liver tissue from mice from both the NASH (Fig. 4A) and the fibrosis studies (Fig. 4B). Several markers showed a significant decrease in expression following treatment in one or both studies. When compared to the vehicle, IFN-γ expression was significantly reduced after I-BET151 treatment in the NASH study as well as in the 5 and 15 mg/kg I-BET151-treatment groups in the fibrosis study (*p* < 0.05, one way ANOVA followed by Fisher LSD test). TNF-α and MCP-1 showed a downward trend towards lower expression levels compared to vehicle in the fibrosis study. Among the fibrosis markers tested, TIMP-1 showed a consistent significant decrease in all three I-BET151 treated groups in the fibrosis study (*p* < 0.05, one way ANOVA followed by Fisher LSD test), and COL1A1 showed a suggestive, but non-significant decrease following treatment in both the NASH and fibrosis studies. Additional results from the follow-up NASH study are shown in Supplementary Table S6.

### RNA-Seq expression/pathways

To better understand the broader transcriptomic changes underlying NASH progression and the effect of I-BET151 on disease-related processes, we performed RNA-seq on the collected liver tissue from healthy, vehicle-treated STAM, and I-BET151-treated STAM mice from the NASH study (i.e. dosed from week 6–9). Comparing healthy to the vehicle, we found 1,922 significantly differentially expressed genes (DEGs; FDR-adjusted *p* ≤ 0.05), representing transcriptomic changes that occur during disease onset in this model. The changes in expression between healthy and STAM mice were overall concordant with the transcriptional signature from one of the human NASH studies from our CMAP analysis, with a significant overlap between the top up- and down-regulated genes between the two species (up-regulated: p = 0.007, odds ratio = 1.5; down-regulated: p = 0.05, odds ratio = 1.7; Fisher’s Exact Test; see Methods), suggesting that the STAM model captures the general transcriptional characteristics of human NASH. To gain an understanding of the underlying functional modulation that the disease-induced DEGs might represent, we performed gene ontology (GO) and pathway analyses using the mouse DEG list. GO analysis showed an enrichment of functions related to immune response, lipid metabolism, inflammation, oxidation-reduction, and cell movement/adhesion. Over 45% (1,094) of the DEGs were also annotated as being membrane-associated or membrane-spanning, suggesting substantial changes in cell signaling and/or extracellular interactions might be playing a role during disease progression in these mice. Pathway analysis using MetaCore (Thomson Reuters) identified several pathways where these DEGs were overrepresented, with the top four most enriched pathways for DEGs including those related to liver inflammation (“Chemokines in inflammation in adipose tissue and liver in obesity, type 2 diabetes and metabolic syndrome X”), cell adhesion (“Cell adhesion: Integrin inside-out signaling in neutrophils” and “Role of cell adhesion in vaso-occlusion in Sickle cell disease”), and Th17 cell migration (“Common mechanisms of Th17 cell migration”).

**Figure 5:**
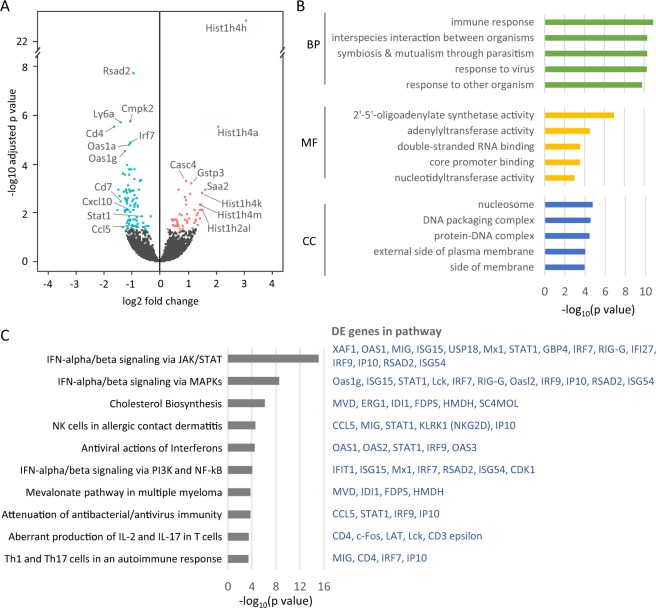
RNA-seq analysis of transcriptional changes after I-BET151 treatment. **(A**) Volcano plot showing the results of the differential expression analysis between the STAM-vehicle and STAM-I-BET151 groups. Genes that were significantly higher after I-BET151 treatment are shown in pink; genes that were significantly lower are shown in blue (FDR-adjusted p < 0.1; statistical analysis by DESeq2). **(B**) Top GO terms of the significant differentially expressed genes (DEGs) from the biological process (BP), molecular function (MF), and cellular component (CC) categories. **(C)** Top pathways enriched in the significant DEGs. Pathways are from MetaCore. The DEGs found each pathway, as reported by MetaCore, are listed to the right, with down-regulated genes (lower after treatment) shown in blue.

We next compared the transcriptional signature of the STAM vehicle group to the group treated with I-BET151. The number of DEGs following I-BET151 intervention was relatively small, even at a relaxed *p* value threshold of 0.1, with only 97 genes that increased in expression after treatment and 141 genes that decreased (FDR-adjusted *p* ≤ 0.1; Fig. 5A). Among the most greatly increased genes were the glutathione transferase *Gstp3* and several histone subunits, including *Hist1h4h*, *Hist1h4a*, *Hist1h4k*, *Hist1h4m*, and *Hist1h2al*. The genes with the greatest decrease following treatment included a striking number of genes with immune-related functions, including *Rsad2*, *Ly6a*, *Cd4*, *Cd7*, *Cxcl10* (also called IP-10), *Stat1*, and *Ccl5*. In agreement with this, the DEGs were strongly enriched for immune-related GO terms, with a more moderate enrichment for external plasma membrane and nucleosome terms (Fig. 5B). Pathway analysis showed significant enrichment and downregulation of several pathways related to IFN-α/β signaling, as well as the “Cholesterol Biosynthesis” pathway (Fig. 5C).

**Figure 6:**
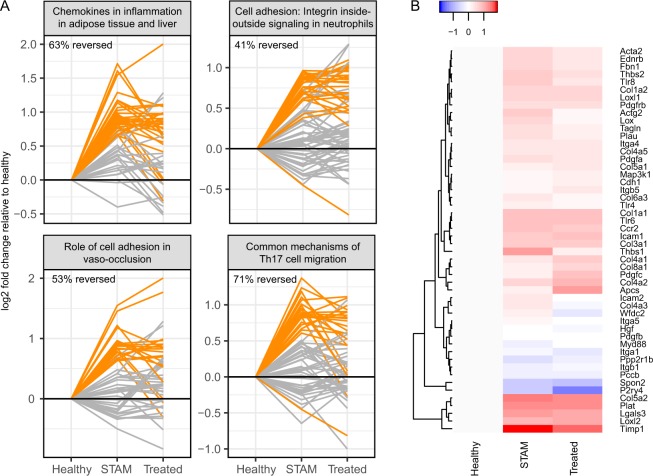
Effect of I-BET151 treatment on disease progression pathways and markers. **(A)** For each of the top four pathways that were altered in the disease state (based on the comparison of healthy vs. STAM-vehicle mice), the progression of expression changes of the pathway genes are shown from Healthy to STAM-Vehicle (STAM) to STAM-I-BET151 10 mg/kg (I-BET151) mice. Each line in the graph shows a single gene, with genes that were significantly altered in STAM vs. healthy highlighted in orange. The percent of the orange genes that were reversed after treatment (i.e. returned towards the healthy state) is indicated for each pathway. **(B)** Heatmap showing expression changes of fibrotic markers in Healthy, STAM-Vehicle, and I-BET151 10 mg/kg treated mice. Expression is shown relative to the Healthy mice.

To determine the effect of I-BET151 treatment directly on disease-relevant processes in the STAM model, we examined how genes in the top four disease-perturbed pathways (i.e. those that changed from healthy to vehicle, described above) changed after treatment (Fig. 6A). In all but one of these pathways, I-BET151 treatment reversed the majority of the significantly up- and down-regulated pathway genes from the disease state, restoring these genes towards the levels observed in healthy livers. The most pronounced reversal was seen in the Th17 migration pathway, in which 71% of the DEGs in disease were reversed after treatment (Fig. 6A, lower right). We also examined expression changes in markers of fibrosis, which may indicate NASH severity and progression. Most of these markers were upregulated in the vehicle relative to healthy, suggesting an increase in fibrosis-related processes during disease onset. Importantly, following I-BET151 treatment these markers showed a decrease in expression relative to the vehicle, suggesting a possible halt in fibrotic processes (Fig. 6B). Among the genes that showed this reversal of expression following treatment were *Timp1*, *Acta2*, *Lox*, *Tlr4*, and *Tlr9*. It should be noted, however, that the histopathology was inconclusive regarding the fibrotic state of the animals used for this part of the study. Comparing again to the human NASH transcriptional signature from the CMAP analysis, we found significant overlap between genes up-regulated during human NASH and genes down-regulated by I-BET151 in the STAM mice—i.e. a reversal of the human disease signature (p = 0.0004, odds ratio = 2.5; Fisher’s Exact Test; see Methods). Altogether, these results suggest that I-BET151 treatment reverses human-relevant NASH-induced genes and pathways back towards healthy levels, particularly in relation to immune responses.

## Discussion

**Figure 7:**
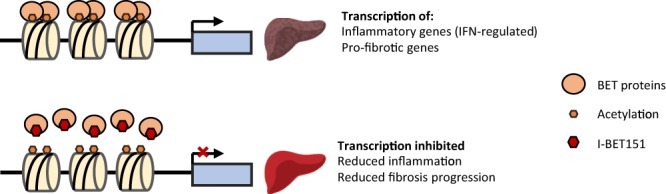
Proposed effects of BET inhibition in NASH. In the disease state, BET protein binding to acetylated histones promotes transcription of inflammatory and pro-fibrotic genes. Upon inhibition of BET protein binding by I-BET151, transcription of these genes is reduced, leading to a corresponding reduction in hepatic inflammation and fibrotic progression.

The current study was undertaken to follow up on a computational prediction by CMAP analysis that BET inhibition could have therapeutic potential for NASH. To test this hypothesis, we examined the effects of I-BET151 in an established STAM mouse model at two distinct time windows: 6–9 weeks to study the effects during NASH onset, and 9–12 weeks to study the effects on fibrosis. We found that therapeutic intervention with I-BET151 significantly improved human-relevant histological and gene expression-based measures of disease severity, including NAS (which is routinely used to diagnose human clinical samples), fibrosis score, fibrosis marker expression (including TIMP1), and inflammatory gene expression (including IFN-γ). A major emphasis of our study was the transcriptomic characterization of the STAM model with I-BET151 intervention through RNA-sequencing of liver tissues to comprehensively identify expression changes during NASH onset and after I-BET151 treatment. This characterization revealed—for the first time in this model system, to the best of our knowledge—that I-BET151 intervention results in the down-regulation of numerous immune-related genes, particularly those involved in interferon signaling. Additionally, I-BET151 treatment resulted in the reversal of fibrosis markers and disease pathway genes towards healthy levels of expression, including genes induced during human NASH. Altogether, these results support our initial computationally-driven hypothesis and suggest that BET inhibition demonstrates therapeutic potential for NASH and other fibrotic diseases (Fig. 7).

### Reduction of glucose dysfunction

Abnormal glucose metabolism is a common phenotype observed in NASH, and there appears to be a strong link between NASH and T2D, with 44% of NASH patients estimated to have diabetes^5^. Linking these two diseases is the phenomenon of insulin resistance, which is known to promote lipolysis and lipogenesis and is key driver of hepatic steatosis^28^. Drugs that improve insulin sensitivity and glucose homeostasis are therefore of considerable interest in the context of NASH, some of which are already being considered as possible treatments for NASH (e.g. PPAR agonists)^28^. Here, we observed reduced serum glucose levels in the mice following IBET-151 treatment relative to vehicle controls in both the NASH and fibrosis studies, suggesting a possible overall improvement in glucose homeostasis. This finding is in agreement with a previous study using I-BET151 in the NOD mouse model of diabetes^29^, where it was demonstrated that treatment of NOD mice with I-BET151 for two weeks led to prolonged normoglycemia, preventing the development of NOD diabetes for 18 weeks. I-BET151 treatment in that study appeared to have a dual mechanism of action, both inducing the pancreatic macrophage population to adopt an anti-inflammatory phenotype (primarily via the NF-κB pathway), and promoting β-cell numbers^29^. Although glucose levels do not inform directly on NASH severity or improvement, these observations from our study and others suggest an additional axis of benefit of I-BET151 treatment for NASH patients that have diabetes. There could also be indirect benefits for other NASH patients, whereby the improvement of glucose homeostasis decreases fatty acid oxidation and thus reduces the effects of lipid accumulation, resulting in a protective effect against NASH progression.

### Anti-inflammatory effects

Inflammation is a central feature distinguishing NASH from NAFLD and is a major driver of progressive fibrotic remodeling of the liver. Clinical studies targeting genes associated with pro-inflammatory pathways, such as apoptosis signaling kinase-1 (ASK1) and C-C motif chemokine receptor 2 (CCR2)^30–32^, have been shown to improve NASH and control the progression of fibrosis, and there is great interest in the identification of additional inflammatory targets for potential therapies. Here, we found that the overall histological improvement in liver sections following I-BET151 treatment was driven by reduced lobular inflammation and hepatocyte ballooning rather than reduced steatosis, suggesting that in this model I-BET151 has a primarily anti-inflammatory effect. This is in line with previous work linking BET inhibition with potent suppression of pro-inflammatory pathways^33^. Interestingly, the specific genes and pathways affected by BET inhibition have been found to be highly cell type specific^33^ indicating that an accurate evaluation of the potential therapeutic impact of BET inhibition for NASH requires examining its effects in liver tissue. Through our RNA-seq analysis of I-BET151 treated STAM livers, we observed a marked decrease in inflammatory gene expression in interferon-related pathways, suggesting suppression of interferon signaling may be a key mechanism of anti-inflammatory action of I-BET151 in this system. BET inhibition has been shown to suppress interferon-stimulated gene expression in the past^34,35^ but the suggestion that this pathway could be a key avenue of effect in liver tissue specifically—at least in this model system—is novel. This finding is intriguing, as interferon signaling has already been implicated in NAFLD and NASH by multiple studies and has been suggested as a possible therapeutic target^36^. In addition, several of the interferon-related genes we observed with significantly decreased expression following I-BET151 treatment have been independently implicated in NASH. For example, the pro-inflammatory chemokine CXCL10 has been shown to induce inflammation and regulate lipogenesis and oxidative stress in NASH^37^, and circulating CXCL10 levels are an independent risk factor for NASH and associated with the severity of lobular inflammation^38^. Another example is STAT1, which is up-regulated in the hepatocytes of NASH patients and has also been shown to be correlated with disease severity, specifically with the NAFLD activity score^39^. We note that from the available data, we are unable to definitively conclude whether the observed pathway changes are primarily induced by reduction in type I, II, or III interferons, since many of the DEGs from these pathways can be modulated by more than one type (e.g. STAT1). Overall, our results demonstrate that BET inhibition in STAM mouse liver tissue has an overall beneficial effect on inflammation and suppresses key inflammatory genes associated with NASH progression and severity.

### Effects on fibrosis

In NASH patients, untreated fibrosis leads to cirrhosis and is associated with increased risk of cancer, need for liver transplant, and mortality. Therefore, treatments that can halt or reverse fibrotic liver injury are of particular need. Key drivers of progressive fibrosis include persistent inflammation and hepatic stellate cell (HSC) activation which result in chronic expression of pro-fibrotic genes involved in e.g. extracellular matrix remodeling. Targeting of these pathways present promising avenues for treatment. Here, we found that multiple dosing concentrations of I-BET151 showed significant anti-fibrotic effects as evidenced by reduction of histological fibrosis score and reduced expression levels of TGFβ and TIMP1. We also saw an overall reduction in the expression of a panel of other fibrotic marker genes based on RNA-seq. These results are in agreement with a previous study in the CCl_4_ mouse model of hepatic fibrosis, where it was found that three BET inhibitors—I-BET151, JQ1, and PFI-1—all decreased pro-fibrotic gene expression in the presence and absence of TGF-β1^18^. Further follow-up with JQ1 in this model also suggested that it could significantly decrease HSC activation and halt or even reverse fibrosis based on histological markers^18^. Although we did not examine reversal of fibrosis in the current study, our findings extend these previous reports of the beneficial effects of BET inhibition on fibrosis to the STAM model, which more closely mirrors human NASH progression.

An important remaining area of work is determining the mechanism of effect of BET inhibition on fibrosis progression in NASH. Given the key role of inflammation in this process, we hypothesize that the decreases in inflammation and interferon signaling we observed following I-BET151 treatment are at least partially responsible. In support of this, a recent mouse steatohepatitis model study revealed that IFN-γ deficiency reduces hepatic fibrosis^40^ suggesting a role specifically for interferon modulation. We also found some evidence for a decrease in HSC activation, including a general decrease in expression of HSC activation markers such as ACTA2, ACTG2, LOX, and TIMP1, although not all of these effects were significant. Previous work also supports a direct mechanism of BET inhibition on pro-fibrotic gene expression: BRD4 was found to be enriched at active enhancer elements associated with known profibrotic transcription factors, and the predicted target genes were enriched for profibrotic functions^18^. More work will be needed to identify the precise mechanisms underlying the effects of BET inhibition on fibrosis, but altogether, our results lend further support to the idea that BET inhibition may be an effective treatment for fibrosis in NASH.

### Limitations and future directions

Understanding a complex disease such as NASH in model systems with therapeutic intervention requires a significantly large sample size. While our study was able to identify inflammatory and fibrotic markers, studies such as ours might suffer from lack of significant statistical power to identify a comprehensive set of inflammatory and fibrosis markers and driver mechanisms due to the limited sample size. Nonetheless, the sample size was adequate to preliminarily evaluate the potential of this compound for treating NASH and fibrosis. The effects of I-BET151 on additional parameters, such as intrahepatic triglycerides, oxidative stress markers, or antioxidant function markers, should also be explored further.

It is important to note that the conclusions of this study, while promising, are inherently limited by the animal model used, and therefore must be interpreted with caution before translating them for human therapeutic use. The STAM model is a chemically (STZ)-induced NASH model which may or may not completely recapitulate features of human NASH that are associated with the metabolic syndrome (e.g., obesity, insulin resistance), and we did not investigate the effects on mice treated with STZ or high fat diet alone. In addition, feasible intervention points will need to be defined for human NASH, which may differ from what was studied here; we focused on treatment starting at the onset of NASH and onset of fibrosis, but later intervention points may be necessary in the human case. Another limitation was that, while transcriptomics characterization revealed that I-BET151 intervention resulted in significant anti-inflammatory and anti-fibrotic activity, the underlying mechanisms that led to these findings (e.g. immune response, regulation of cholesterol biosynthesis, interferon signaling, and effects on hepatocytes, Kupffer cells, HSCs) still remain to be determined. Further elucidation of these mechanisms will require significant additional work outside of the scope of this pilot study, and will be an important focus for future research across the field.

## Conclusions

Overall, the observed reductions in NAS and fibrosis score support the potential therapeutic application of BET inhibition for NASH and fibrotic liver diseases, and suggest that BET inhibition exerts a primarily anti-inflammatory effect on liver tissue, likely via interferon pathways (Fig. 7). Further clinical investigations could help evaluate the therapeutic potential of BET inhibition for NASH patients. To the best of our knowledge, there are currently no publicly available RNA-seq datasets profiling human NASH; the availability of such human data in a large cohort would likely greatly advance our understanding of the disease, potential translatability of the current model, and allow for validation of the findings from this and other mouse model studies. Finally, this study adds to the growing number of other promising molecular targets^30,41^ that are currently being evaluated for this life-threatening disease.

## Materials and Methods

### Experimental design

The objective of this study was to test the hypothesis that BET inhibition has therapeutic potential for NASH. To achieve this, the STAM mouse model of NASH was used to evaluate the effects of the BET inhibitor I-BET151 *in vivo*. The overall design of the experiment is shown in Fig. 1. Sample sizes were chosen based on a power analysis of a pilot study using normal mice and STAM mice treated with Telmisartan or vehicle. Endpoints and data collection timepoints were pre-defined based on prior knowledge of the STAM model. No animals were excluded during the course of the study. Animals were assigned randomly to treatment groups and investigators were blinded to the group assignments of the animals during the experiment, including the two certified pathologists performing histopathology assessments. Outlier data points were defined using standard criteria on a per-assay basis, as described in the text.

### nSTZ-HFD induced STAM NASH mouse model

The animal studies described here were carried out at Wuxi AppTec, 90 Delin Rd, Shanghai, China on behalf of GlaxoSmithKline (GSK). During these studies, all animal procedures were conducted in accordance with the GSK Policy on the Care, Welfare and Treatment of Laboratory Animals and were reviewed the Institutional Animal Care and Use Committee either at GSK or by the ethical review process at the institution where the work was performed. The experimental protocol for this study was approved by the GSK office of Animal welfare, Ethics and Strategy and Wuxi AppTec Co. Shanghai, China.

The STAM NASH mouse model was obtained from Shanghai Lingchang Laboratory Animal Co. LTD. The STAM model is created by a combination of chemical (streptozotocin) and dietary interventions consisting of high fat diet *ad libitum* in C57BL/6 mice^22^. STAM mice develop NASH with hepatocyte fat accumulation and inflammation at about week 7–9, and advance to fibrosis with nodule formation around week 11–12.

Briefly, 2 day old male C57BL/6 mice were injected by a single subcutaneous injection of 200 μg of streptozotocin to cause islet destruction and then fed a high fat diet (60% energy from fat, D12492; Research Diets, Inc) starting at 4 weeks of age and continuing for the entire duration of each study. The study design for each of the NASH and fibrosis studies are shown in Fig. 1.

At the termination of each study, animals were sacrificed by exsanguination through direct cardiac puncture under ether anesthesia. Serum and liver samples were collected for biomarker detection and histopathological analysis. Study end points included body and liver weights, plasma biochemistry, glucose, triglycerides (TG), alanine aminotransferase (ALT), aspartate aminotransferase (AST), total cholesterol (TCHO), and mRNA expression of inflammatory tumor necrosis factor-α (TNF-α), monocyte chemoattractant protein-1 (MCP-1), interferon-γ (IFN-γ), fibrotic transforming growth factor-β (TGF-β), collagen type 1 (COL1A1), and TIMP metallopeptidase inhibitor 1 (TIMP-1) genes. Liver sections were cut from FFPE blocks and stained with Hematoxylin and eosin stain (H&E) or Sirius Red to assess NAFLD score and liver fibrosis respectively.

### Treatment Groups

The test compound GSK1210151A (I-BET151) was developed at GSK^21^. For this study, I-BET151 was prepared as a suspension in 0.5% (w/w) hydroxypropyl methylcellulose (HPMC) with 0.1% (w/w) Tween 80 to a final concentration of 5 mL/kg. Dosing was administered orally once daily at 5 mg/kg, 10 mg/kg or 15 mg/kg. The vehicle control was prepared using 1.0% (w/v) MethylCellulose (MC). For positive control, Telmisartan, an angiotensin II receptor antagonist (QW32M-GS), was purchased from Tokyo chemical industry CO. LTD. The stock solution of Telmisartan was prepared in a suspension containing 1.0% (w/v) MC to a final concentration of 5 mL/kg and dosed orally once a day at 10 mg/kg.

### Experimental Protocol(s)

#### Body and Liver Weights

Body weight for each mouse was measured daily before and during the treatment period. In addition, body and liver weights were recorded after the animals were sacrificed.

#### Serum Glucose, Insulin, Triglycerides, Cholesterol, and Aminotransferases

Blood/plasma samples were collected from cardiac puncture at the termination of the study. Plasma levels of glucose, ALT, AST, TG and TCHO were measured using an auto biochemical analyzer Type 7180 (HITACHI CO., Ltd. 1-6-6, Tokyo). The kits used in the analysis were obtained from DiaSys Diagnostic Systems GmbH (#: 141 2707170 1 for ALT; 141 2607170 1 for AST; 112 1307170 1 for TCHO; 112 5717170 1 for TG; 141 251 71701 for glucose). Insulin was measured by ELISA kit EZRMI-13K (Millipore, USA).

#### Liver Cholesterol and Triglycerides

Liver TG levels were determined using a commercial kit purchased from BioVision Co. LTD (155 S. Milpitas Blvd., Milpitas, CA 95035 USA.CAT. #K622-100). Liver TCHO levels were determined using a cholesterol quantitation kit purchased from Sigma-Aldrich Co. LLC (CAT. # MAK043).

#### Gene expression of Inflammatory and Fibrosis Markers

Total RNA was extracted from each liver sample using RNeasy Mini Kit (Qiagen-74104). Extracted RNA was reverse transcribed to cDNA using a High Capacity cDNA Reverse Transcription Kit (AB-4368814).

Real-time PCR for each gene was performed using Faststart Universal Probe Master (ROX) (Roche-04914058001). Relative expression of target genes was compared after normalization against Rplp0 reference gene. The qPCR cycling conditions were: 95 °C 10 min followed by 40 cycles of 95 °C 15 s (Melt) and 60 °C 1 min (Anneal/Extend). qPCR measurements were carried out using the Bio Rad-CFX Connect system. The gene expression of the target gene in each test sample was determined by relative quantification using the comparative Ct (ΔΔCt) method. This method measured the Ct difference (ΔCt) between target gene and internal reference gene (18S) and then compared the ΔCt values of treated samples to naïve group samples.

Liver pathological study: NAS score and Fibrosis score: For H&E staining, four micron thick sections were cut from Paraffin-embedded blocks (Leicia RM2235) of liver tissue obtained from the left lateral lobe and stained with Lillie-Mayer’s H&E solution (Leicia Autostainer XL). Oil-red staining was performed on 15 micron thick sections obtained from the O.C.T blocks (Leicia CM1950).

To visualize collagen deposition, fixed liver sections were stained using picro-Sirius red solution. NAFLD Activity score (NAS) was calculated according to Kleiner’s criteria as previously described^27^. Scoring using NAS was performed by two certified pathologists blinded to treatment group using the three criteria of steatosis, hepatocyte ballooning, and lobular inflammation, and a third in-house pathologist was included in the final assessment of NAS scores. Steatosis in hepatocytes was scored as 0, 1, 2, or 3 if there were less than 5%, 5–33%, 33–66%, and greater than 66% hepatocytes with fat, respectively. Hepatocyte ballooning was scored as 0 if there was none, 1 if there were few ballooned cells, and 2 if there were many cells with prominent ballooning. Lobular inflammation was scored as 0, 1, 2, or 3 if there were none, less than 2, 2–4, or greater than 4 inflammatory foci per 200x field, respectively. These individual scores were summed to give the NAS for each animal.

### Connectivity Map (CMAP)

Transcriptional response profiles for GSK compounds were generated on a high-throughput gene expression profiling technology L1000 platform^42^ for over 400 compounds, some at multiple doses, in 11 cell lines. The assay measures the expression of 978 carefully selected landmark transcripts and infers the expression of the remaining 19 K transcripts using a computational model trained on tens of thousands of historical gene expression profiles^43,44^. Initial data processing was performed directly on the crude cell lysates in a 384-well plate format at the Genometry facility (http://www.genometry.com) using their standard pipeline. Briefly, intensity measurements for each sample were evaluated first to assess if the relative expression of control genes are consistent. All samples that passed well and plate level thresholds were scaled, normalized and log transformed. In the final step, we use a robust z-scoring procedure to generate differential expression values from normalized profiles.

Disease expression signatures were generated from NextBio. Samples were grouped by the tissue of origin, study ID and disease categorization. Only tissues with at least 3 normal and 3 disease samples were considered for further processing. Altogether, nearly 6000 disease signatures from human clinical disease samples were generated from NextBio. Each disease signature consists of 500 most up-regulated probe sets and 500 most down-regulated probe sets selected by fold change between disease samples and normal samples.

### RNA-Seq and Data Analysis

Total RNA was isolated from liver samples of healthy, STAM-vehicle, and STAM-I-BET151 mice (n = 4 for each group) upon termination of the study using Trizol with phase separation and a Qiagen miRNeasy kit with DNase digestion. Sample libraries were prepped on the Tecan using an Illumina TruSeq mRNA stranded kit with 1 μg input, 7 min fragmentation, and 10 PCR cycles. Libraries were quantified using a Kapa qPCR kit and QuantStudio and then sequenced on an Illumina HiSeq. 2500 with 101 bp paired end reads to a depth of over 100 million reads per sample.

Reads were mapped using STAR v.2.5.1b^45^ with default parameters. Uniquely mapped reads were used as input to gene quantification using htseq-count. Gene counts were adjusted for library size using DESeq2^46^ and then this package was used to identify differentially expressed genes between healthy and vehicle-treated STAM and then between vehicle-treated STAM and I-BET151-treated STAM. One STAM-I-BET151 treated sample was excluded during this analysis due to low gene counts. Pathway analysis was performed using MetaCore (Thomson Reuters), using all DEGs as input to identify overrepresented pathways compared to a background of all expressed genes in the study. Markers for fibrosis were obtained from a mouse fibrosis (CCL4) model following treatment with a BRD4 inhibitor JQ4^18^.

Data from a human NASH study was obtained from GEO (GSE48452). The transcriptional signature from this study was one that came up as a significant hit for BET compounds in our CMAP analysis (Supplementary Table S1). After removing genes that could not be mapped between mouse and human, we extracted the top 500 genes with the largest positive and negative log2 fold changes in the human dataset (250 positive and 250 negative) based on the healthy vs. NASH patient cohort comparison. We then compared these lists with the set of genes with a log2 fold change of ≥0.5 or ≤−0.5 in the mouse healthy vs. STAM-vehicle comparison to identify genes with a concordant direction of change in both studies. We also compared the positive fold change human list with the set of genes with a log2 fold change of ≤−0.5 in the mouse STAM-vehicle vs. STAM-I-BET151 comparison to identify human-relevant genes that were reversed by I-BET151 in the mouse model. Fisher’s exact test was used to assess the significance of overlap in each comparison, taking into account the full set of genes considered in each study (excluding those that could not be mapped between human and mouse).

### Statistical analysis

Differences between individual groups for the biometric data were assessed using one way ANOVA followed by Fisher LSD test. NAS and fibrosis scores were analyzed using non-parametric Wilcoxon test. Results are expressed as mean ± standard error mean (SEM) and p values of <0.05 were considered statistically significant.

## Electronic supplementary material

Supplementary Information

## Data Availability

Data generated for this study are available through the Gene Expression Omnibus (GEO, accession no. GSE114261).
