# Supplementary material for: BET Inhibition Improves NASH and Liver Fibrosis
Source: Sci Rep. 2018 Nov 22;8:17257. doi: 10.1038/s41598-018-35653-4 (PMC6250695; doi:10.1038/s41598-018-35653-4)
Supplement: Supplementary file 1 — Supplementary Information [file 41598_2018_35653_MOESM1_ESM.pdf]

Supplementary Information

BET Inhibition Improves NASH and Liver Fibrosis

Sarah A. Middleton, Neetu Rajpal, Leanne Cutler, Palwinder Mander, Inmaculada Rioja, Rab K. Prinjha, Deepak Rajpal, Pankaj Agarwal, Vinod Kumar

GEO Study ID	Tissue Origin	Disease	Source	Gene Signature	Study Details	Normal Samples	Disease Samples	Pvalue	Specificity	Enrichment	Signatures with better specificity
GSE48452.3	Human	Steatosis	NextBio	Livers from steatosis patients vs healthy controls	Ahrens M, Ammerpohl O, von Schönfels W, Kolarova J et al. DNA methylation analysis in nonalcoholic fatty liver disease suggests distinct disease-specific and remodeling signatures after bariatric surgery. Cell Metab 2013 Aug 6;18(2):296-302. PMID: 23931760	14	14	0.0269	0.1944	-0.762	191
GSE48452.1	Human	NASH	NextBio	Livers from non-alcoholic steatohepatitis patients vs healthy controls	Ahrens M, Ammerpohl O, von Schönfels W, Kolarova J et al. DNA methylation analysis in nonalcoholic fatty liver disease suggests distinct disease-specific and remodeling signatures after bariatric surgery. Cell Metab 2013 Aug 6;18(2):296-302. PMID: 23931760	14	18	0.003	0.0087	-0.9498	12
GGSE24807.1	Human	NASH	NextBio	Liver biopsy from 7-18 y old non-alcoholic steatohepatitis patients vs healthy controls	Liu W, Baker SS, Baker RD, Nowak NJ et al. Upregulation of hemoglobin expression by oxidative stress in hepatocytes and its implication in nonalcoholic steatohepatitis. PLoS One 2011;6(9):e24363. PMID: 21931690	5	12	0.0155	0.2984	-0.8048	415
GSE25583.3	Mouse	Liver Fibrosis	NextBio	Liver of wild type mice after fibrosis induction by CCL4 treatment-2wk vs 0 day	Marquardt JU, Seo D, Gómez-Quiroz LE, Uchida K et al. Loss of c-Met accelerates development of liver fibrosis in response to CCl(4) exposure through deregulation of multiple molecular pathways. Biochim Biophys Acta 2012 Jun;1822(6):942-51. PMID: 22386877	4	4	0.0001	0.0593	-0.9666	13
GSE25583.4	Mouse	Liver Fibrosis	NextBio	Liver of wild type mice after fibrosis induction by CCL4 treatment-3wk vs 0day	Marquardt JU, Seo D, Gómez-Quiroz LE, Uchida K et al. Loss of c-Met accelerates development of liver fibrosis in response to CCl(4) exposure through deregulation of multiple molecular pathways. Biochim Biophys Acta 2012 Jun;1822(6):942-51. PMID: 22386877	4	4	0.0002	0.1135	-0.9518	27

Table S1. Enrichment, Pvalue and Specificty results obtained from the application of the Connectivity Map to BET compounds
Enrichment score is a non-parametric Kolmogorov Smirnov (KS) statistic obtained by computing the ranks for a set of compound instances from the list of all instances for a disease signature of interest. The scores range from +1 to -1. P-value is an estimate of the likelihood that the enrichment of a set of compound instances from the list of all compound instances for a disease signature of interest would be observed by chance. This value is determined by performing the enrichment of 100000 sets of instances selected at random from the set of all instances.

Specificity is an estimate of the uniqueness of the connectivity between a set of compound instances and a disease signature of interest. It is based upon the results generated for that set of instances across 6000 diverse disease signatures. High specificity scores indicate the connectivity found between the set of compound instances and the signature of interest is not exceptional and suggests that it may be involved in multiple biological events.

Sample	Healthy	Vehicle	IBET-151 (10 mg/kg)	Telmisartan (10 mg/kg)
Glucose (mmol/L)	8.62 ± 0.42	28.63 ± 0.74	23.71 ± 0.60 *	32.00 ± 0.85
Serum Triglycerides (mmol/L)	1.03 ± 0.08	1.22 ± 0.10	1.08 ± 0.09	1.20 ± 0.14
Serum Cholesterol (nmmol/L)	2.28 ± 0.07	2.95 ± 0.08	3.97 ± 0.28	3.40 ± 0.10
ALT (u/L)	25.8 ± 0.87	77.50 ± 4.19	68.7 ± 9.94	68.70 ± 6.28
AST (uL)	154.0 ± 24.29	228.60 ± 12.86	205.9 ± 19.47	250.10 ± 28.55
Liver Weight (g)	0.93 ± 0.02	0.89 ± 0.02	0.82 ± 0.02	0.83 ± 0.02
Liver Triglycerides (ug/mg)	152.00 ± 24.83	62.92 ± 5.76	70.38 ± 13.99	22.67 ± 2.35
Liver Cholesterol (ug/mg)	1.38 ± 0.14	1.07 ± 0.05	1.26 ± 0.14	0.886 ± 0.08
Body Weight (g)	23.2 ± 0.44	18.0 ± 0.50	17.7 ± 0.48	17.0 ± 0.26
Liver/Body Weight (g)	0.043 ± 0.002	0.02 ± 0.004	0.06 ± 0.008	0.07 ± 0.006

Table S2. Serum/liver biomarkers and body/liver weights in the NASH study.
Data expressed as mean ± SEM. *p < 0.05 vs. vehicle control. All data points measured at termination. Average ALT levels and average AST levels in the early intervention. ALT, alanine aminotransferase; AST, aspartate aminotransferase.

Sample	Healthy	Vehicle	IBET-151 (5 mg/kg)	IBET-151 (10mg/kg)	IBET-151 (15 mg/kg)	Telmisartan (10 mg/kg)
Glucose(mmol/L) (Day 1 of dosing)	5.4 ± 0.20	20.4 ± 0.39	20.7 ± 0.55	19.3 ± 0.52	18.2 ± 0.93	18.4 ± 0.76
Glucose (mmol/L)	3.0 ± 0.22	22.3 ± 1.22	17.5 ± 1.92 *	16.9 ± 0.88 *	16.4 ± 1.46 *	20.2 ± 1.84
Insulin (ng/ml) (Day 1 of dosing)	0.9 ± 0.06	0.2 ± 0.03	0.3 ± 0.04	0.3 ± 0.07	0.2 ± 0.04	0.2 ± 0.04
Insulin (ng/ml)	0.3 ± 0.05	0.4 ± 0.05	0.3 ± 0.04 *	0.3 ± 0.07	0.3 ± 0.02 *	0.5 ± 0.04
Serum Triglycerides (mmol/L)	1.23 ± 0.04	3.5 ± 0.72	7.9 ± 3.25 *	6.5 ± 2.37	12.4 ± 4.97 *	5.0 ± 1.63
Serum Cholesterol (nmmol/L)	2.30 ± 0.05	5.1 ± 0.68	5.5 ± 0.75	5.6 ± 0.43	6.5 ± 2.37	5.5 ± 0.44
ALT (u/L)	21 ± 1.00	43.4 ± 2.38	44.8 ± 6.39	48.8 ± 7.27	50.8 ± 6.89	44.6 ± 3.93
AST (uL)	145 ± 2.00	143.6 ± 7.10	182.3 ± 26.22	207.3 ± 32.46	197.5 ± 29.02	140.9 ± 16.59
Liver Weight (g)	1.02 ± 0.02	1.03 ± 0.02	1.06 ± 0.05	0.95 ± 0.06	0.95 ± 0.04	0.81 ± 0.02 *
Liver Triglycerides (ug/mg)	74.77 ± 4.95	45.69 ± 7.77	54.59 ± 9.84	49.81 ± 9.18	75.97 ± 11.75 *	32.09 ± 8.66
Liver Cholesterol (ug/mg)	2.42 ± 0.09	2.92 ± 0.12	2.74 ± 0.14	2.90 ± 0.19	3.15 ± 0.17	2.99 ± 0.17
Body Weight (g)	24.4 ± 0.31	17.0 ± 1.03	17.2 ± 0.88	16.4 ± 0.58	16.5 ± 0.86	14.9 ± 0.99
Liver/Body Weight (g)	0.042 ± 0.001	0.061 ± 0.003	0.062 ± 0.003	0.058 ± 0.004	0.058 ± 0.003	0.056 ± 0.003

Table S3. Serum/liver biomarkers and body/liver weights in the fibrosis study.

Data expressed as mean ± SEM. *$p < 0.05$ vs. vehicle control. All data points measured at termination of study unless otherwise noted. Average ALT levels and average AST levels in the early intervention. ALT, alanine aminotransferase; AST, aspartate aminotransferase.

Sample	Healthy	Vehicle	IBET-151 (5 mg/kg)	IBET-151 (10mg/kg)	IBET-151 (15 mg/kg)	Telmisartan (10 mg/kg)
Glucose(mmol/L) Day 1	9.00 ± 0.43	29.36 ± 0.51	30.19 ± 0.53	29.93 ± 0.91	29.19 ± 0.60	30.3 ± 0.70
Glucose (mmol/L) Day 22	7.37 ± 0.42	22.15 ± 1.33	19.73 ± 1.07	24.10 ± 2.08	24.56 ± 1.35	25.2 ± 2.06
Insulin (ng/ml) Day 22	0.86 ± 0.17	0.49 ± 0.02	0.55 ± 0.05	0.40 ± 0.01	0.51 ± 0.06	0.60 ± 0.06
Serum Triglycerides (mmol/L)	1.95 ± 0.10	1.92 ± 0.06	2.50 ± 0.21	3.91 ± 1.24	12.74 ± 6.74 *	2.54 ± 0.29
Serum Cholesterol (nmmol/L)	2.78 ± 0.07	4.22 ± 0.23	4.49 ± 0.16	5.67 ± 0.60	7.43 ± 1.80 *	4.50 ± 0.14
ALT (u/L)	31.67 ± 3.65	88.07 ± 11.86	54.83 ± 3.34	75.17 ± 8.32	90.40 ± 19.07	72.4 ± 8.23
AST (uL)	227.43 ± 65.00	251.40 ± 20.69	211.43 ± 21.78	236.50 ± 31.58	274.60 ± 71.65	194.1 ± 15.6
Liver Weight (g)	0.94 ± 0.04	0.90 ± 0.04	0.83 ± 0.02	0.88 ± 0.05	0.84 ± 0.04	0.76 ± 0.04
Liver Triglycerides (ug/mg)	54.00 ± 2.47	44.67 ± 5.40	40.29 ± 6.93	60.94 ± 6.21	71.56 ± 6.92 *	27.7 ± 3.84
Liver Cholesterol (ug/mg)	2.92 ± 0.08	3.34 ± 0.11	3.16 ± 0.14	3.25 ± 0.18	3.49 ± 0.08	3.39 ± 0.16
Body Weight (g)	21.7 ± 0.44	15.5 ± 0.58	14.8 ± 0.36	14.6 ± 0.91	14.8 ± 1.18	14.4 ± 0.33
Liver/Body Weight (g)	0.043 ± 0.002	0.002 ± 0.004	0.07 ± 0.057	0.057 ± 0.084	0.084 ± 0.000	0.004 ± 0.052

Table S4. Serum/liver biomarkers and body/liver weights from a second followup NASH study (6 -9 weeks).
Data expressed as mean ± SEM. *$p < 0.05$ vs. vehicle control. All data points measured at termination. Average ALT levels and average AST levels in the early intervention. ALT, alanine aminotransferase; AST, aspartate aminotransferase.

Column1	Score	Healthy	Vehicle	IBET-151 (5 mg/kg)	IBET-151 (10 mg/kg)	IBET-151 (15 mg/kg)	Telmisartan (10 mg/kg)
		N=7	N=15	N=7	N=6	N=5	N=7
**Steatosis**	**0**	7			1		2
	**1**		12	7	4	3	5
	**2**		1		1	2	
	**3**		2				
**Lobular inflammation**	**0**	6			1		
	**1**	1	5	6	3	3	2
	**2**		7	1	2	1	4
	**3**		3			1	1
**Hepatocyte ballooning**	**0**	7	3	4	3	3	2
	**1**		9	3	3	1	5
	**2**		3			1	
**NAS (mean ± SD)**		0.14 ± 0.14	4.20 ± 0.48	2.57 ± 0.30 *	2.67 ± 0.61 *	3.60 ± 1.03	3.29 ± 0.36

Table S5. Comparison of NAS from a second followup NASH study (6-9 weeks).
Experimental design is the same as NASH study described in Fig. 1 but with the addition of multiple IBET-151 dose concentrations (5, 10, and 15 mg/kg). * = $p < 0.05$ compared to vehicle.

Sample	Healthy	Vehicle	IBET-151 (5 mg/kg)	IBET-151 (10mg/kg)	IBET-151 (15 mg/kg)	Telmisartan (10 mg/kg)2
TNF-α	0.90 ± 0.15	3.68 ± 0.39	1.90 ± 0.18 **	3.8 ± 0.61	2.20 ± 0.43	2.35 ± 0.33 *
Collagen1A1	1.04 ± 0.11	3.29 ± 0.39	2.20 ± 0.22	3.0 ± 0.43	2.60 ± 0.36	3.00 ± 0.31
MCP1	0.92 ± 0.12	4.76 ± 0.63	2.28 ± 0.31 **	5.78 ± 1.39	4.14 ± 1.01	3.46 ± 0.30
TGF-β1	1.02 ± 0.08	1.64 ± 0.03	0.84 ± 0.19 **	1.23 ± 0.23	1.24 ± 0.13	1.40 ± 0.09
TIMP-1	0.90 ± 0.04	6.30 ± 0.23	2.84 ± 0.35 **	4.63 ± 0.77	4.08 ± 0.76	4.96 ± 0.79
IFN-γ	0.87 ± 0.16	1.85 ± 0.06	0.71 ± 0.09 **	0.92 ± 0.14 **	0.75 ± 0.35**	1.21 ± 0.19 *

Table S6. Comparison of biomarker expression from a second followup NASH study (6-9 weeks).
Experimental design is the same as NASH study described in Fig. 1 but with the addition of multiple IBET-151 dose concentrations (5, 10, and 15 mg/kg). * = $p < 0.05$ compared to vehicle.

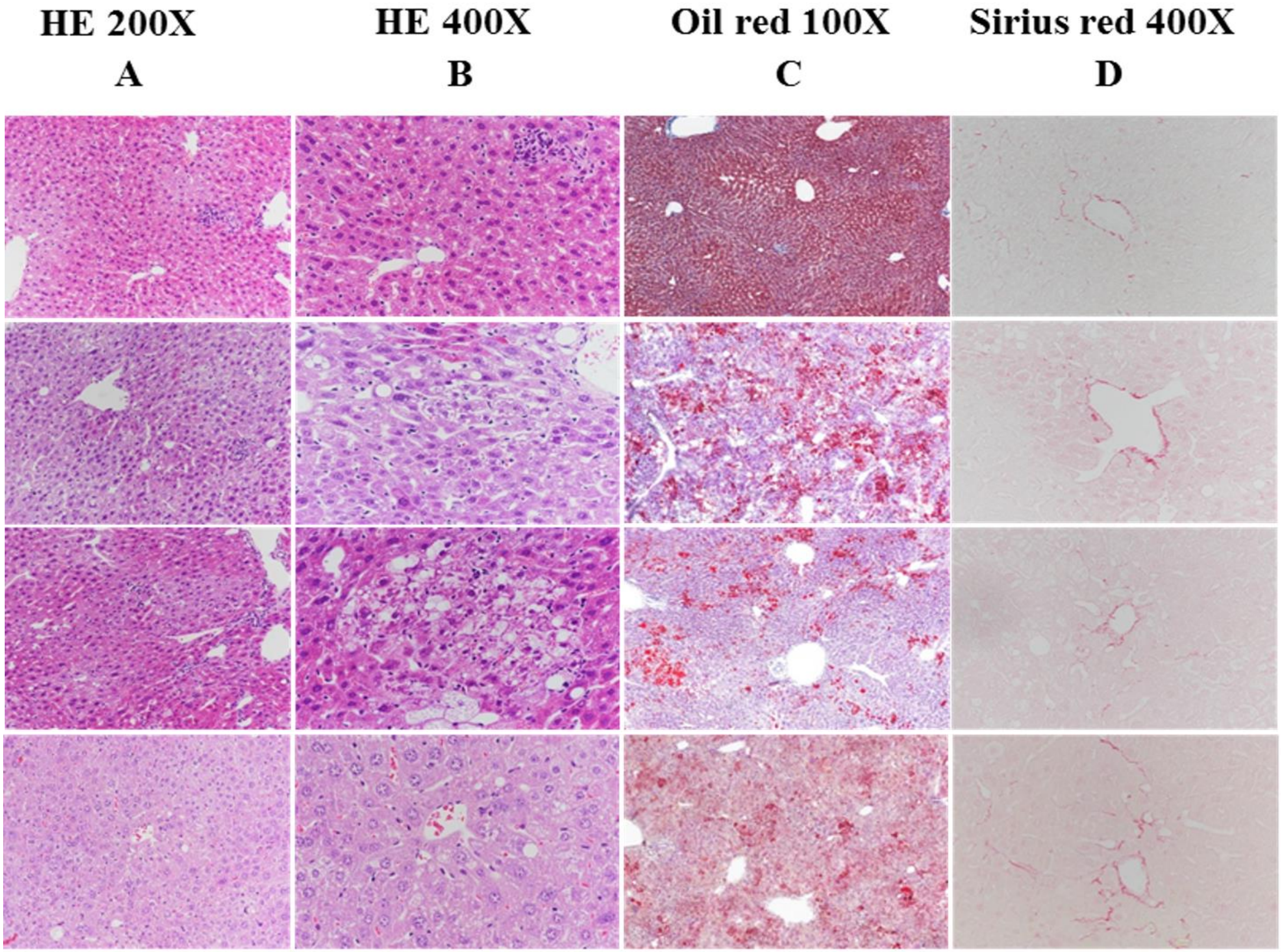

Figure S1: Enlarged microscopic images from the NASH study. H&E stained (200x and 400x), Oil-red (100x) stained, and Sirius-red (400x) stained liver sections from healthy (row 1), vehicle (row 2), telmisartan (row 3), and I-BET151 (row 4) treated NASH STAM mice collected at week 9.

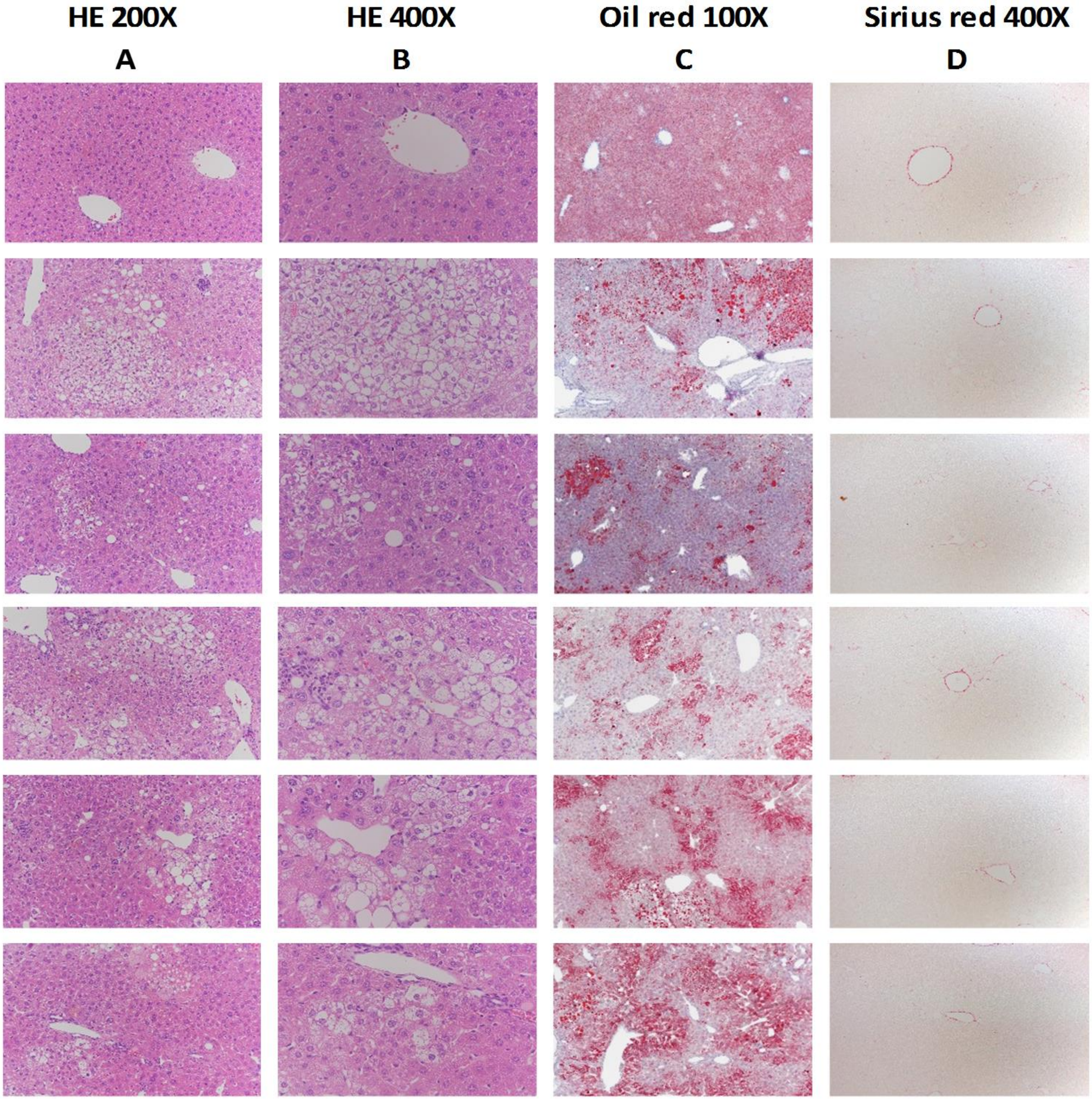

HE 200X
A
HE 400X
B
Oil red 100X
C
Sirius red 400X
D

Figure S2: Enlarged microscopic images from the fibrosis study. H&E stained (200x and 400x), Oil-red (100x) stained, and Sirius-red (400x) stained liver sections from healthy (row 1), vehicle (row 2), telmisartan (row 3), I-BET151 5mg/kg (row 4), I-BET151 10mg/kg (row 5), and I-BET151 15mg/kg (row 6) treated NASH STAM mice collected at week 12.
